# Automatic Estimation of the Interference Subspace Dimension Threshold in the Subspace Projection Algorithms of Magnetoencephalography Based on Evoked State Data

**DOI:** 10.3390/bioengineering11050428

**Published:** 2024-04-26

**Authors:** Ruochen Zhao, Ruonan Wang, Yang Gao, Xiaolin Ning

**Affiliations:** 1 Institute of Large-Scale Scientific Facility and Centre for Zero Magnetic Field Science, Beihang University, Beijing 100191, China; zhaoruochen@buaa.edu.cn (R.Z.); by1917076@buaa.edu.cn (R.W.); 2 School of Instrumentation Science and Optoelectronic Engineering, Beihang University, Hangzhou 310051, China; yanggao@buaa.edu.cn; 3 National Institute of Extremely-Weak Magnetic Field Infrastructure, Hangzhou 310051, China; 4 Shandong Key Laboratory for Magnetic Field-Free Medicine and Functional Imaging, Shandong University, Jinan 310051, China

**Keywords:** magnetoencephalography, interference suppression, subspace projection, subspace dimension, optically pumped magnetometer

## Abstract

A class of algorithms based on subspace projection is widely used in the denoising of magnetoencephalography (MEG) signals. Setting the dimension of the interference (external) subspace matrix of these algorithms is the key to balancing the denoising effect and the degree of signal distortion. However, most current methods for estimating the dimension threshold rely on experience, such as observing the signal waveforms and spectrum, which may render the results too subjective and lacking in quantitative accuracy. Therefore, this study proposes a method to automatically estimate a suitable threshold. Time–frequency transformations are performed on the evoked state data to obtain the neural signal of interest and the noise signal in a specific time–frequency band, which are then used to construct the objective function describing the degree of noise suppression and signal distortion. The optimal value of the threshold in the selected range is obtained using the weighted-sum method. Our method was tested on two classical subspace projection algorithms using simulation and two sensory stimulation experiments. The thresholds estimated by the proposed method enabled the algorithms to achieve the best waveform recovery and source location error. Therefore, the threshold selected in this method enables subspace projection algorithms to achieve the best balance between noise removal and neural signal preservation in subsequent MEG analyses.

## 1. Introduction

Magnetoencephalography (MEG) is a neuroimaging technique with a high spatiotemporal resolution [1], which has been applied in the localization of epilepsy and early diagnosis research on psychiatric diseases [2,3]. With the development of optically pumped magnetometers (OPMs) in recent years, wearable OPM-MEG systems [4,5] have not only overcome the disadvantages of traditional MEG devices [6] that need to operate at low temperatures and sensors that are fixed and far away from the subject’s body but have also acquired an improved level of signal sensitivity [7]. Therefore, OPM-MEG systems have broad prospects in neuroscience research and clinical applications [8,9]. However, because MEG signals are very weak, even if the measurements are performed in a magnetically shielded room (MSR) [10,11], environmental noise interference is significantly stronger than neural signals [12]. Therefore, noise suppression algorithms are crucial for MEG signal processing [13].

Widely used MEG interference suppression algorithms can be divided into two categories: regression methods [14,15] based on reference sensors, and subspace projection algorithms. Although the principle of the regression algorithm is simple and easy to implement, it cannot eliminate interference that is not recorded by the reference sensors, and the complexity of the background magnetic field makes accurate modeling difficult [13]. Hence, in this study, we focus on optimizing subspace projection algorithms. These algorithms divide internal neural signals and external interference signals by projecting MEG signals onto internal and external subspaces [16]. The interference subspace is usually defined by the eigenvectors corresponding to the top x largest singular values in the noise singular value matrix. Therefore, the dimension of the interference subspace, which is the value of x, is an important threshold in subspace projection algorithms. If the threshold is set too low, the interference subspace will not be able to capture sufficient noise, and the noise suppression effect will not be adequate; however, if the threshold is set too high, some internal neural signals will be falsely projected into the interference subspace, resulting in signal distortion [16]. Algorithms based on subspace projection can be implemented in various signal domains, such as signal space projection (SSP) [17] and signal space separation (SSS) [18] in the space domain; common temporal subspace projection (CTSP) [19], spatiotemporal signal space separation (tSSS) [20], and dual-signal subspace projection (DSSP) [21] in the time domain; and spectral signal space projection (S3P) in the frequency domain [22]. Although the performance of these algorithms has been widely verified, it is necessary to select appropriate thresholds according to the noise levels of different data to achieve a balance between the denoising effect and signal distortion.

Some studies have estimated the thresholds of the subspace projection algorithms. The S3P algorithm removes noise peaks (spectral spikes) by expressing the noise dimension through the eigenvalues of the signal cross-spectral density matrix; however, this may ignore noise that is broadly distributed over the full frequency band [22]. Refs. [23,24] estimated the appropriate truncation values of the spherical harmonic functions for the SSS algorithm in OPM-MEG by simulation and iterative computation. However, these methods are only applicable to estimating the parameters of the SSS; they are difficult to apply to all subspace projection algorithms. In addition, because the actual value of the neural signal is not available from the measurement data, Ref. [25] investigated the effects of different thresholds in the CTSP algorithm on the denoising effect and signal distortion using the standard deviation of the signal to represent the noise level and the neural signal represented by the alpha rhythm frequency band. However, because there is an unknown proportion of noise in the frequency band in addition to the neural signal, a more accurate method for describing the neural signal is needed. In other studies, suitable thresholds were estimated only by manual experience, such as observing the variation of the waveform and spectrum between different thresholds. Thus, to the best of our knowledge, there is no automatic estimation method for the interference subspace dimension threshold that applies to most subspace projection algorithms. The threshold selected by the method proposed in this paper enables the subspace projection algorithm to achieve the best balance between noise suppression and signal distortion reduction.

Therefore, we propose a method to automatically estimate suitable thresholds for the subspace projection algorithms using MEG evoked state data, so that the subspace projection algorithms can accurately and quickly find suitable thresholds to process data with different noise levels. The degrees of noise suppression and signal distortion are designed as two objective functions, and the optimal threshold within the selected range is solved using the weighted-sum method. The effectiveness of the proposed method is verified through simulation, somatosensory-evoked and auditory-evoked experimental data using the classical subspace projection algorithm. The threshold selected by the method proposed in this paper enables the subspace projection algorithm to achieve the best balance between noise suppression and signal distortion reduction.

This paper is structured as follows. In Section 2, we present the principle of the proposed threshold estimation method, and we describe the design scheme and parameter settings of the experiment. Section 3 presents the specific experimental results, and a detailed discussion is presented in Section 4. Finally, we conclude the paper in Section 5.

## 2. Materials and Methods

In this section, we first define the signal model and illustrate the importance of the external subspace dimension in the algorithm using the principles of the SSP algorithm. Then, we describe how the method proposed in this study selects the appropriate dimension threshold. Finally, we describe our experimental design.

### 2.1. Subspace Projection Algorithm

We denote the total signal *Y* obtained from the measurement as the sum of the neural signal *B* inside the brain and the external interference signal *N*, which, after denoising, corresponds to $\hat{Y}$, $\hat{B}$, and $\hat{N}$:

(1) Y=B+N,

(2) $$\hat{Y}=\hat{B}+\hat{N},$$

Subspace projection algorithms focus on removing external environmental noise and mostly ignore sensor noise.

The SSP algorithm is a classical subspace projection algorithm in the space domain [17], where *N* is estimated using empty-room noise data. We apply singular value decomposition to the empty-room data matrix *N*:

(3) $$N=\left[f_{1},\cdots ,f_{n}\right]\left[\begin{array}{cccc} \lambda_{1} & 0 & \cdots & 0\\ 0 & \lambda_{2} & \cdots & 0\\ \vdots & \vdots & \ddots & \vdots \\ 0 & 0 & \cdots & \lambda_{n} \end{array}\right]\left[\begin{array}{c} g_{1}^{T}\\ \vdots \\ g_{n}^{T} \end{array}\right],$$

where $\lambda_{1},\lambda_{2}\cdots \lambda_{n}$ are singular values and $f_{1},\cdots ,f_{n}$ are the corresponding spatial singular vectors. Among the *n* singular values, we choose the vector of the top-*x* singular values that are considerably larger than the other singular values. These vectors form the orthogonal bases of the interference subspace. We define the matrix $F_{x}=\left[f_{1},\cdots ,f_{x}\right]$ and extract the projection operator of the interference subspace $F_{x}F_{x}^{T}$. After removing the interference, the internal neural signal $\hat{B}$ is obtained by projecting *Y* onto the internal subspace orthogonal to the external subspace:

(4) $$\hat{B}=Y\left(I-F_{x}F_{x}^{T}\right),$$

where *x* is the dimension of the interference subspace, which is an important factor for determining the denoising effect and the degree of signal distortion of the SSP. Similar thresholds are also present in other subspace projection algorithms [19,21,22]. However, it is usually set based on manual experience by observing singular values; therefore, in the next subsection, we design a method to estimate this threshold automatically.

### 2.2. Threshold Estimation Algorithm

A suitable threshold should enable the subspace projection algorithm to remove most of the noise while retaining as much of the neural signal as possible and reducing signal distortion. The threshold estimation problem can be modeled as a multi-objective optimization problem using the degree of noise suppression and the degree of distortion of the neural signal as the two objective functions [26]. In addition, according to previous studies, the scope of the parameter should not be too wide (less than 10); therefore, it is sufficient to use a simple traversal method. The key is to design the two objective functions in a reasonable manner.

First, the degree of neural signal distortion can be defined as the ratio of the neural signals before and after denoising using Equations (1) and (2), respectively:

(5) $$\frac{\hat{B}}{B}=\frac{\hat{Y}-\hat{N}}{Y-N},$$

The exact neural signal is not known for the MEG signal; hence, we adopt the definitions of noise and neural signals of interest from the time–frequency analysis of the evoked state data [27]. In event-related evoked data, we typically have time segments (e.g., the 100 ms post-stimulus component) and frequency bands (e.g., alpha rhythms) of interest; thus, the total signal *Y* can be defined as the total power value of that part of the signal. Noise *N* is defined as the average power in the pre-stimulus time segment (normally called the baseline period) that does not contain any significant neural signals as expressed by Equations (6) and (7):

(6) $$Y\left(f,t\right)=\frac{1}{n}\sum_{k=1}^{n}{\left|y_{k}\left(f,t\right)\right|}^{2},$$

(7) $$N\left(f\right)=\frac{1}{nm}\sum_{k=1}^{n}\sum_{t^{\prime }=a}^{0}{\left|y_{k}\left(f,t^{\prime }\right)\right|}^{2},$$

Here, *f* and *t* denote the specific frequency band and time segment of interest, respectively; *n* denotes the total number of trials; and *m* is the number of sampling points in the baseline segment. We define the neural signal as the absolute value of the difference in the multichannel MEG data [27]. According to Equation (5), the objective function $F_{B}$ of the degree of distortion of the neural signal is thus defined as:

(8) $$F_{B}=\frac{\hat{B}}{B}=\frac{\frac{1}{l}\sum_{i=1}^{l}\;\left|{\hat{Y}}_{i}-{\hat{N}}_{i}\right|}{\frac{1}{l}\sum_{i=1}^{l}\;\left|Y_{i}-N_{i}\right|},$$

where *l* is the number of channels. Conversely, the degree of noise suppression can be expressed as the proportion of noise in the signal before and after denoising. Usually, the magnitude of the total signal is very large, even after bandpass filtering, and is ten times larger than the evoked MEG signal, which is only approximately hundreds of fT and contains considerably more noise than the neural signal. Therefore, we simplify the noise to the total signal power:

(9) $$f_{N}=N\approx \sum_{t=1}^{T}y{\left(t\right)}^{2},$$

In practice, each channel exhibits a different noise level. A small amount of random noise can usually be eliminated by the superposed average; therefore, we add channel weights $w_{i}$ to the objective function $F_{N}$ for the degree of noise suppression to focus on channels with relatively high noise:

(10) $$F_{N}=\frac{N}{\hat{N}}=\frac{\sum_{i=1}^{l}w_{i}f_{N}\left(i\right)}{\sum_{i=1}^{l}w_{i}{\hat{f}}_{N}\left(i\right)},$$

(11) $$w_{i}=\frac{\sigma_{i}}{\sum_{i=1}^{l}\sigma_{i}},$$

where σ is the standard deviation of each channel signal, and the weight is positively correlated with the noise level [28].

After normalizing $F_{B}$ and $F_{N}$ for all thresholds to [0, 1], we obtain the total objective function, $F_{all}$ using the weighted-sum method, which is a commonly used multi-objective optimization method [29]:

(12) $$F_{all}\left(x\right)=\alpha F_{B}\left(x\right)+\left(1-\alpha \right)F_{N}\left(x\right),$$

where *x* is a threshold of the subspace projection algorithm, and α is the weight used to balance the two objective functions. However, in practice, when *x* is larger, $F_{B}$ is considerably smaller than $F_{N}$, and although the signal distortion is already sizeable, $F_{all}$ will still be large because of its linear relationship with $F_{N}$. To render $F_{all}$ more sensitive to serious signal distortion and reduce the impact of larger $F_{N}$ variations on $F_{all}$, we adopt the idea of an activation function, normalize $F_{N}$ to [0, 2], and substitute it into the hyperbolic tangent function for the solution [30]:

(13) $$F_{all}\left(x\right)=\alpha F_{B}\left(x\right)+\left(1-\alpha \right)\tanh\left(F_{N}\left(x\right)\right),$$

**Figure 1:**
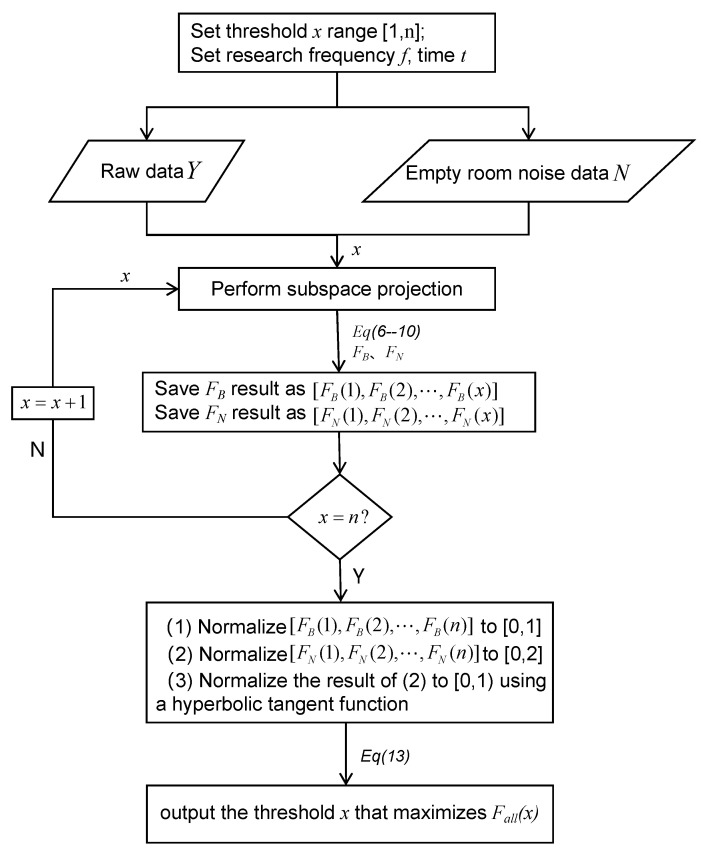
Flowchart of the threshold estimation method.

The threshold that gives the maximum value of $F_{all}$ in the selected range is the optimal solution estimated by the method described in this study. The process and details of this method are depicted in Figure 1. Except the time–frequency transform, the time complexity of the program implementing the proposed method is O(l+N), where *l* is the number of channels and *N* is the selected threshold range.

### 2.3. Experiments

To verify the effectiveness of our proposed threshold estimation method, we tested it using two classical subspace projection algorithms in simulation, somatosensory-evoked and auditory-evoked experimental data.

#### 2.3.1. Simulation Experiment

**Figure 2:**
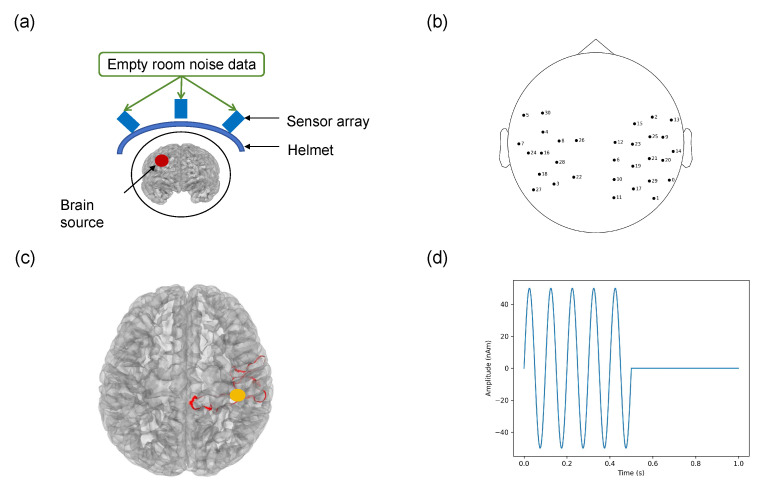
(**a**) Simulation experiment system. (**b**) Top view of the 31 channel sensors relative to the head position. (**c**) Location of simulation neural source. (**d**) Amplitude of the simulation neural source.

A schematic diagram of the simulation system used in this study is presented in Figure 2a. It comprises the internal space of the brain, an array of sensors, and external noise.

The internal brain space was obtained by segmenting the T1-MRI files from a 26-year-old male participant. Thirty-one OPM sensors installed on an 85-channel rigid helmet were simulated as the sensor array [31]. The locations of the sensors on the helmet were optimized for the somatosensory-evoked experiment. A three-dimensional laser scanner (HSCAN Prince 775, Scantech Inc., Hangzhou, China) was used to scan the participant wearing the helmet. Co-registration was performed using the OMMR Toolbox [32] to obtain the position (Figure 2b) of the sensor relative to the brain space.

The total signal is composed of an internal simulation signal and the measured empty-room noise data. To simulate the neural signals of the brain, the source of the internal simulation signal was defined as the current dipole in the internal space of the brain (Figure 2d). The source with a peak amplitude of 50 nA·m and a frequency of 10 Hz was activated in the first 500 ms in a period of 1 s; a total of 300 trials were conducted. To validate the results of the somatosensory evoked experiment, the location of the source was set near the center of the postcentral gyrus (somatosensory cortex) [33]. To avoid random errors in the data processing of a single dipole, 30 points were randomly selected on the cortical surface (yellow area in Figure 2c) with a radius of 3 mm from the center as the origin. We calculated the average value of all points as the final result. The position of the sensor on the helmet was unchanged when measuring the empty-room noise data, and two empty-room noise datasets were measured; one dataset was used with the internal simulation signal to form the raw total signal, and another for the subspace projection algorithms to estimate the noise matrix.

#### 2.3.2. Stimulus-Evoked Experiments

**Figure 3:**
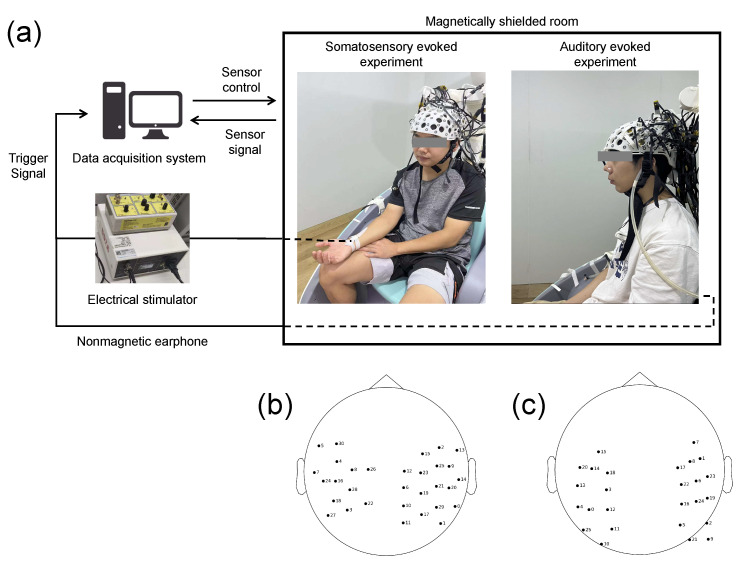
(**a**) System for somatosensory- and auditory-evoked experiments. (**b**) Sensor locations for somatosensory-evoked experiment. (**c**) Sensor locations for auditory-evoked experiment.

To further verify the validity of the method proposed in this study, we tested the data with different noise levels and stimulation modalities, and conducted somatosensory- and auditory-evoked experiments in two MSRs. The experimental system is shown in Figure 3a. No magnetic field compensation coils were present in the MSRs (ambient field amplitude below 13 nT). Our OPM-MEG research protocol was approved by the Ethics Committee of Beihang University, and written informed consent was obtained from all participants.

Somatosensory-evoked experiment: The number and location of the sensors used in the somatosensory-evoked experiment were the same as those used in the simulation experiments (Figure 3b). The subject was the same as that in the simulation experiment. The sensor type is the second-generation OPM sensor (QZFM, QuSpin Inc., Louisville, CO, USA). A commercial electrical stimulator (DS7A, Digitimer Ltd., Welwyn Garden City, UK) was used to stimulate the median nerve of the left hand. The stimulation threshold was set to the current amplitude at which small thumb movements were visible. The stimulation duration was 200 μs, with the interval being 1 s. In total, 300 epochs were performed; 300 s of empty-room data were recorded before the experiment.

Auditory-evoked experiment: In the auditory-evoked experiment, 26 sensors of the same type as for the somatosensory-evoked experiment were used; their positions are shown in Figure 3c. The subject was a 26-year-old right-handed healthy male. In the experiment, we played a pure tone stimulus at 1000 Hz to the left ear of the subject using plastic earphones. The stimulation duration was 20 ms, with the interval being 1 s. In total, 480 epochs were performed; 300 s of empty-room data were recorded before the experiment.

#### 2.3.3. Data Processing

All signal generation and processing codes were executed using the MNE-Python platform [34]. The data sampling rate was 1000 Hz, and the bandpass filter was used to cover the range 1–40 Hz.

In the simulation data, the dipole activation moment was used as the stimulus moment (0 ms), segmenting the −200 to 800 ms data into one epoch. The time segment of the signal of interest was set to 0–500ms, the frequency band was 9–11 Hz, and the noise time segment was −200–0 ms. In the somatosensory-evoked data, the M35 component was used as the signal of interest based on the findings in [35,36], where the electrical stimulation moment was used as the stimulus moment (0 ms), segmenting the −100 to 200 ms data into one epoch. The time segment of M35 was set to 30–50 ms, the frequency band was 5–15 Hz, and the noise time segment was −100–0 ms. In the auditory-evoked data, the M100 component was used as the signal of interest based on the findings in [37], where the pure tone stimulation moment was used as the stimulus moment (0 ms), and the −100 to 300 ms data were segmented into one epoch. The time segment of M100 was set to 90–120 ms, the frequency band was 5–20 Hz, and the noise time segment was −100–0 ms. The bad-epoch rejection threshold was 2000 fT.

We tested the proposed method by selecting two subspace projection algorithms, SSP and S3P. We adjusted the threshold ranges and value intervals for different data and algorithms based on the empty-room noise data. The threshold ranges of the SSP and S3P algorithms were set from 1 to 10 in the simulation and somatosensory-evoked data, and from 1 to 8 in the auditory-evoked experimental data; the value intervals were 1. The balance parameter α in $F_{all}$ was set to 0.5.

#### 2.3.4. Evaluation Metrics

We calculated three quantitative evaluation metrics to assess the results of the experiment: (1) peak error (PE); (2) mean absolute error (MAE); and (3) location error (LE). All calculations were performed on the superimposed average waveforms. $B_{sim}$ and $B_{recon}$ are the simulated neural signal and the reconstructed signal after denoising, respectively. PE (Equation (14)) is used to measure the degree of distortion of the neural signal, and *P* is the number of peak moments of the sine wave of the neural signal. The MAE reflects the degree of deviation of $B_{recon}$ compared to $B_{sim}$ (Equation (15)), where *N* represents the number of time sampling points:

(14) $$\mathrm{PE}=\frac{1}{P}\sum_{i=1}^{P}\left|B_{recon}\left(t_{i}\right)-B_{sim}\left(t_{i}\right)\right|,$$

(15) $$\mathrm{MAE}=\frac{1}{N}\sum_{t=1}^{N}\left|B_{recon}\left(t\right)-B_{sim}\left(t\right)\right|,$$

In the simulation data, LE was defined as the Euclidean distance between the real simulated dipole position and the dipole position of $B_{recon}$ obtained at the peak moment of the signal using the equivalent current dipole (ECD) method [38]. Since somatosensory-evoked and auditory-evoked experiments do not have exact information on the coordinates and magnitude of the neural source, we used minimum norm estimation (MNE) [39] to analyze the localization of sources. In addition, each experiment was performed to compare the results of the average waveforms and time–frequency analyses based on the wavelet transform.

## 3. Results

### 3.1. Simulation Experiment

**Figure 4:**
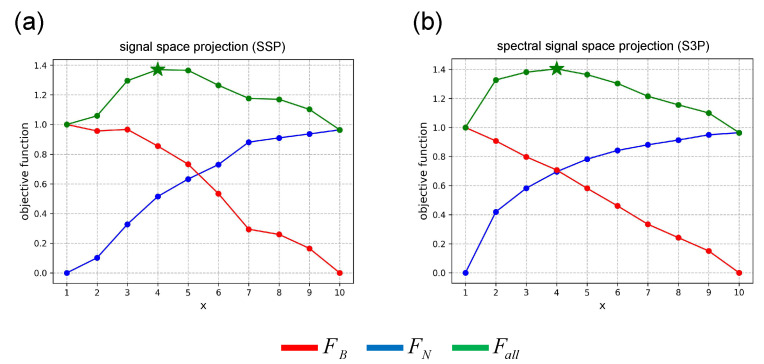
Threshold results in the simulation experiment. (**a**) SSP. (**b**) S3P.

For the simulation experiment, Figure 4 shows the results of the thresholds of the two subspace projection algorithms calculated using the method developed in this study. The red line is the sub-objective function $F_{B}$ representing the degree of signal distortion, the blue line is the sub-objective function $F_{N}$ representing the degree of noise suppression, and the green line is the objective function $F_{all}$ representing the overall effect. It can be seen that *x* = 4 is the best solution for the SSP and S3P algorithms. The optimal solutions selected by the proposed method are collectively referred to as the “optimal threshold”, and the fifth-ranked thresholds are collectively called the “inferior threshold”.

Table 1 shows the results of PE, MAE, and LE for the SSP and S3P algorithms with different thresholds, sorted by the results of $F_{all}$. Owing to space limitations, only the results of the top five thresholds are shown. The optimal threshold selected by the proposed method achieves the best results in all metrics.

**Figure 5:**
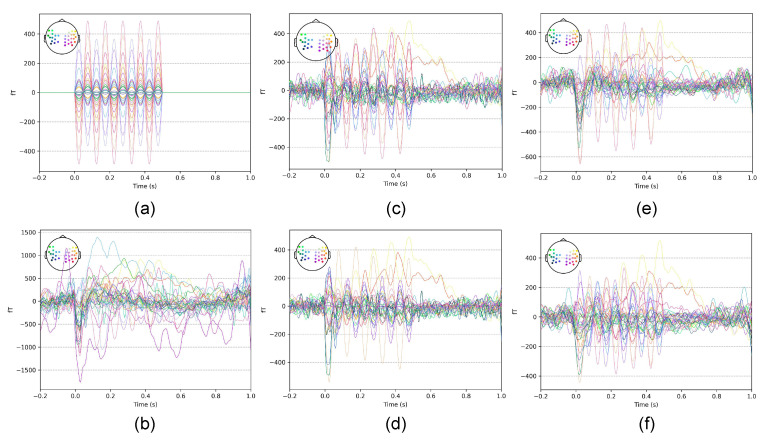
Average waveforms in the simulation experiment with different thresholds. (**a**) Internal simulated signal. (**b**) Bandpass filtering only. (**c**) SSP, *x* = 4. (**d**) SSP, *x* = 7. (**e**) S3P, *x* = 4. (**f**) S3P, *x* = 6.

The average waveforms of the signals from each algorithm after denoising using different thresholds in the simulation experiment are shown in Figure 5. Owing to space limitations and to observe the difference easily, we only show the waveform comparison between the optimal and inferior thresholds; all subsequent results are shown in the same way. SSP and S3P exhibited significantly less signal distortion when using the optimal threshold *x* = 4 (Figure 5c,e) compared to the respective inferior thresholds *x* = 7 and *x* = 6 (Figure 5d,f).

**Figure 6:**
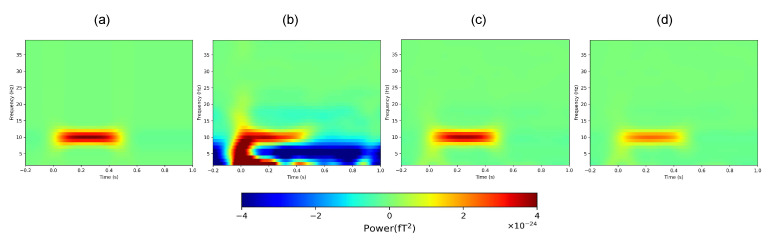
Average results of all channels of time–frequency analysis in the simulation experiment. (**a**) Internal simulated signal. (**b**) Bandpass filtering only. (**c**) SSP, *x* = 4. (**d**) SSP, *x* = 7.

**Figure 7:**
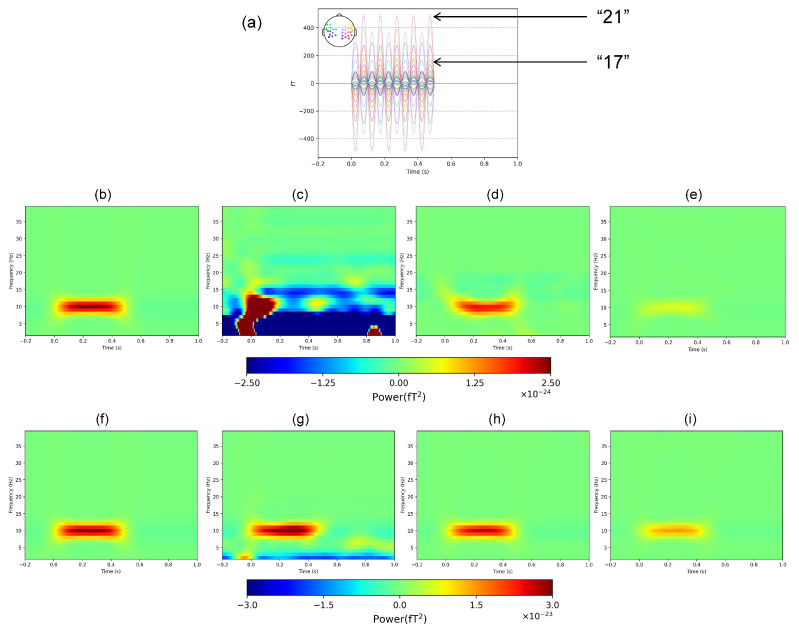
(**a**) Time–frequency analysis results of different channels in the simulation experiment. (**b**–**e**): “17” Channel results. (**b**) Internal simulated signal. (**c**) Bandpass filtering only. (**d**) SSP, *x* = 4. (**e**) SSP, *x* = 7. (**f**–**i**): “21” Channel results. (**f**) Internal simulated signal. (**g**) Bandpass filtering only. (**h**) SSP, *x* = 4. (**i**) SSP, *x* = 7.

The results of the time–frequency analysis are shown in Figure 6 and Figure 7; here, we only show the results of the SSP algorithm. Figure 6 shows the average values of the data from all sensor channels. Figure 6c shows that the optimal threshold not only removes most of the noise but also produces less distortion compared with the inferior threshold, and is more similar to the internal simulated signal.

We also tested the method on two channels with different signal amplitudes. The channel information of “17” and “21” is shown in Figure 7a. Similar to the results in Figure 6, the results in Figure 7 show that the optimal threshold results are closer to the internal simulated signal regardless of the amplitude.

### 3.2. Somatosensory-Evoked Experiment

**Figure 8:**
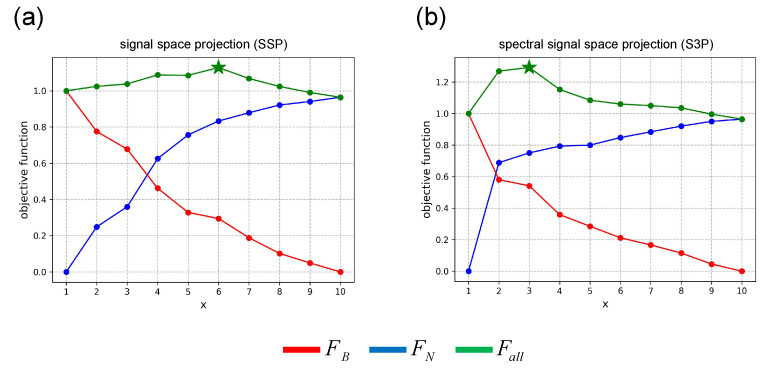
Threshold results in somatosensory-evoked experiment. (**a**) SSP. (**b**) S3P.

Figure 8 shows the results of the thresholds of the three subspace projection algorithms calculated using the method proposed in this study in a somatosensory-evoked experiment. The optimal thresholds for SSP, and S3P are *x* = 6 and *x* = 3, respectively.

**Figure 9:**
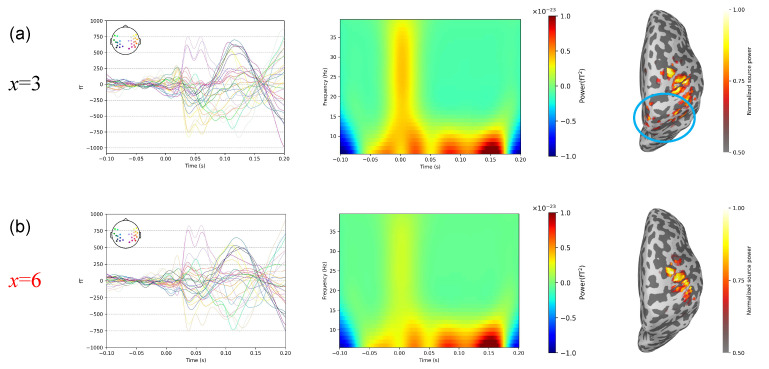
Comparison of results with different thresholds in somatosensory-evoked experiment. (**a**) Results for the inferior threshold, *x* = 3. (**b**) Results for the optimal threshold, *x* = 6. The left row is the result of averaging the superimposed waveforms, the middle row is the result of time–frequency analysis, and the right row is the result of source localization.

As shown in Figure 9, because of space limitations, we consider SSP as an example to compare the results of the average waveforms, time–frequency analysis, and source localization between the optimal threshold (*x* = 6) and the inferior threshold (*x* = 3). Referring to the findings of [33,36], the results of the average waveforms show that the SSP algorithm using the optimal threshold makes the M35 component more obvious (35 ms wave peak). The time–frequency analysis results clearly demonstrate that utilizing the optimal threshold with the SSP algorithm preserves the most M35 signal power while removing additional noise, achieving a better balance between noise suppression and signal distortion. The source activation area of M35 is concentrated in the postcentral gyrus. However, in the source localization result of the inferior threshold, other brain areas (blue circle) are incorrectly activated, which could be caused by noise. In contrast, the source localization result of the optimal threshold is more concentrated and is almost the same as that of the reference.

### 3.3. Auditory-Evoked Experiment

**Figure 10:**
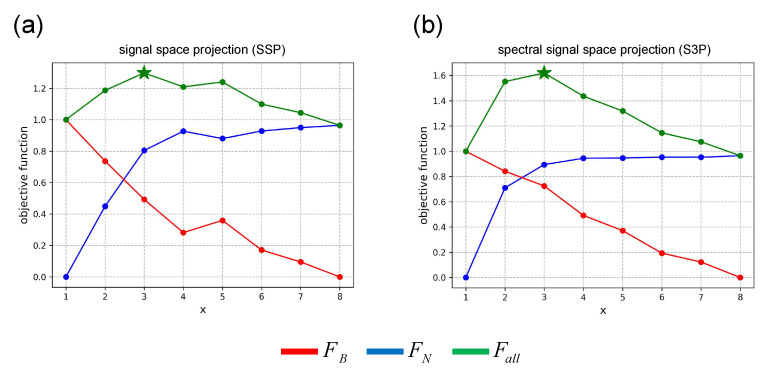
Threshold results in auditory-evoked experiment. (**a**) SSP. (**b**) S3P.

Figure 10 shows the results of the thresholds of the three subspace projection algorithms calculated using the method proposed in this study in a auditory-evoked experiment. The optimal thresholds for SSP and S3P are *x* = 3, respectively.

**Figure 11:**
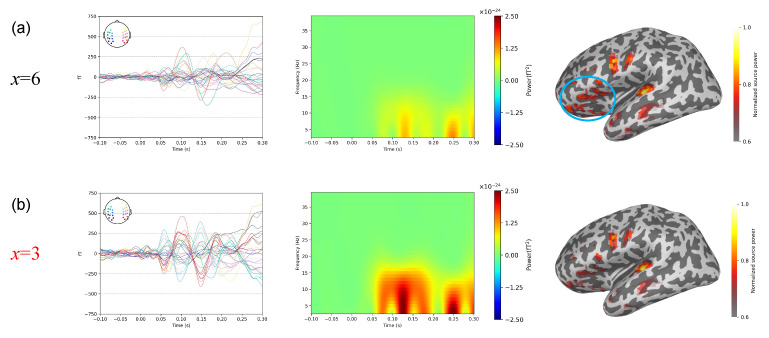
Comparison of results with different thresholds in auditory-evoked experiment. (**a**) Results for the inferior threshold, *x* = 6. (**b**) Results for the optimal threshold, *x* = 3. The left row is the result of averaging the superimposed waveforms, the middle row is the result of time–frequency analysis, and the right row is the result of source localization.

Similar to the somatosensory-evoked experiment, we use the SSP as an example to compare the results between the optimal threshold (*x* = 3) and inferior threshold (*x* = 6). Referring to the findings of [37] as shown in Figure 11, the averaged waveforms and time–frequency analyses demonstrate that the optimal threshold substantially preserves both the waveform and power of M100 with minimal noise interference, contrasting with the inferior threshold, resulting in more pronounced signal distortion. The source activation area of M100 is concentrated in the temporal lobe. Compared with the source localization obtained using the optimal threshold, the results from the inferior threshold exhibit errors in activating additional brain regions (indicated by the blue circle), likely attributable to heightened signal distortion.

## 4. Discussion

Before analyzing MEG signals, it is necessary to remove a large amount of environmental noise interference from the data. The denoising capability of the subspace projection algorithm, one class of classical denoising algorithms, has been widely verified. However, such algorithms typically need to adjust the thresholds to determine the dimensions of the external subspace in different data, and unsuitable thresholds may lead to poor denoising or excessive signal distortion. To the best of our knowledge, most current studies tend to select this threshold subjectively, such as by observing the amplitude and number of noise spikes in the spectrum, or observing the results of averaged waveforms after processing using different threshold, all of which require repeated tests to find the optimal results, making it difficult to automatically select the appropriate threshold for different data. Therefore, the significance of this study lies in the design of a method that can automatically estimate suitable threshold for subspace projection algorithms based on MEG evoked-state data. Our method constructs sub-objective functions to describe the degree of noise rejection and signal distortion, and then obtains the optimal threshold within the selected threshold range using the weighted-sum method. We did not find similar methods of automatically estimating thresholds applicable for multiple subspace projection algorithms, so we did not compare different threshold estimation methods, and our focus was on whether we could find the optimal parameters using the method proposed in this paper. The primary focus of this study is to determine the parameter threshold enabling the subspace projection algorithm to achieve its optimal performance. It does not entail enhancements to the subspace projection algorithms themselves. The proposed method applies to most subspace projection algorithms in principle. In addition to the SSP and S3P algorithms tested in this paper, the time-related thresholds used for signal separation in algorithms such as CTSP and tSSS can also be estimated using our method [19,20].

The SSS series algorithms are also a class of classical subspace projection algorithms; however, they were not tested in this study because the SSS usually requires a large amount of sensor data (greater than 150 channels) to construct an accurate model of the spherical harmonic functions. There are some SSS algorithm optimization methods for OPM-MEG with fewer sensors, which also rely on multi-axis sensors to increase the number of channels and are only applicable to SSS [23,24]. Limited by our experimental conditions, using the SSS algorithm on a system with only thirty pure radial OPM sensors was not effective. In future work, we will increase the number of sensors or use multi-axis sensors to test our method. However, unlike other algorithms that have only one parameter, more parameters can affect the performance of the SSS algorithm, including the position of the origin, truncation values of the spherical harmonic functions. Because the approach in this study uses a traversal method to find the threshold of the parameter, the time cost of finding the optimal thresholds in the SSS algorithm is high. In the future, we will investigate more efficient threshold optimization methods to solve this problem. Homogeneous field correction [40] is a newly proposed denoising algorithm for OPM-MEG systems that can remove most of the homogeneous noise interference. However, we did not test it because it does not require setup thresholds. Moreover, the ability of the method to suppress nonhomogeneous interference needs to be further investigated; it may be possible to extend the space-temporal domain similar to that in eSSS [41,42], which is a worthwhile line of future work.

For the objective function, we designed a weight value α to balance the degree of noise suppression and signal distortion, and in the practical test, we chose 0.5, which is equivalent to ignoring this weight, although better results were achieved. This is because we considered the effect of the two sub-objective functions on the waveform results and source localization to be a complex issue. Thus, further research is needed in our subsequent work to clarify how to automatically and reasonably set this balanced weight value for different studies (brain network analysis, signal classification, etc.). On this basis, we also need to investigate whether an objective optimization method that is more effective than the weighted sum method exists. In addition, the threshold range in this study must be set manually, although the results prove that when the threshold range contains all reasonable thresholds, the threshold selected by our method is the optimal threshold within the range. However, this method can only be considered semi-automatic in terms of threshold selection. Therefore, it is necessary to investigate the automatic selection of an appropriate threshold range for different types of data.

Owing to the limitations of the experimental conditions, we mainly tested the OPM-MEG system with only a few sensors. Other systems that need to apply the subspace projection algorithm (e.g., SQUID) can also use the method proposed in this paper to determine the threshold; however, it is necessary to set up a suitable threshold range. In addition, the signals of interest in the objective function can be set in the appropriate time–frequency bands according to different studies. The method in this study is concerned with the signal power in the time–frequency band; subsequently, we will also investigate the phase response and other aspects.

Overall, our threshold estimation method enables us to select an optimal threshold for the subspace projection algorithm that can suppress the most noise interference without causing significant distortion of the neural signal.

## 5. Conclusions

In this paper, we proposed a method for automatically estimating the interference subspace dimension threshold in the subspace projection algorithm, by selecting the threshold that enables the subspace projection algorithm to best balance noise suppression and signal distortion when denoising MEG data. Our method obtains the neural signals of interest and noise signals in specific time–frequency bands by performing time–frequency transformations on evoked state data, and uses them to construct the objective functions for the degree of noise suppression and signal distortion. By summing them with weights, the optimal threshold within the given range was obtained by traversal. Finally, the validity of our method was verified with three classical subspace projection algorithms using simulation, somatosensory-evoked, and auditory-evoked experimental data.

## Figures and Tables

**Table 1:** Performance of subspace projection algorithms with different thresholds in simulation experiment.

Method	Threshold	$F_{\mathrm{all}}$	PE (fT)	MAE (fT)	LE (mm)
SSP	*x* = 4	1.369	137.03	47.25	2.80
	*x* = 5	1.365	138.54	49.56	3.57
	*x* = 3	1.295	149.75	51.25	3.32
	*x* = 6	1.264	155.05	52.11	4.93
	*x* = 7	1.175	159.04	54.77	5.48
S3P	*x* = 4	1.403	152.15	55.11	2.93
	*x* = 3	1.380	177.29	56.00	4.47
	*x* = 5	1.363	188.26	59.29	3.05
	*x* = 2	1.337	212.77	59.20	5.15
	*x* = 6	1.302	201.89	59.71	5.57

## Data Availability

The data presented in this study are available on request from the corresponding author. The data are not publicly available due to the privacy policies of the Ethics Committee of Beihang University.
